# Organelle-specific hydrogen sulfide metabolism governs redox homeostasis to regulate plant autophagy and cadmium stress resilience

**DOI:** 10.1016/j.redox.2026.104177

**Published:** 2026-04-17

**Authors:** Reyes Carrillo, Inmaculada Moreno, Angeles Aroca, Cecilia Gotor

**Affiliations:** Instituto de Bioquímica Vegetal y Fotosíntesis. Consejo Superior de Investigaciones Científicas and Universidad de Sevilla, Avenida Américo Vespucio, 49, Seville, 41092, Spain

**Keywords:** Arabidopsis, Autophagy, Cadmium stress, Hydrogen sulfide, Persulfidation, Reactive oxygen species, Redox balance, Sulfenylation

## Abstract

Hydrogen sulfide (H_2_S), traditionally regarded as a toxic threat to the environment and living organisms, is now considered an important gasotransmitter that helps regulate redox balance and autophagy in plants. However, how different sources of H_2_S in certain organelles help control stress responses remains unclear. In our study, how H_2_S is handled in various subcellular compartments of *Arabidopsis thaliana* was investigated in depth using null mutants defective in H_2_S production (*des1, sir, cas-c1, str1* and *str2*) or consumption (*oas-a1, oas-b* and *oas-c).* Under normal physiological conditions, disruption of H_2_S homeostasis in any cellular compartment altered endogenous H_2_S levels and induced basal autophagy. These findings suggest that maintaining a specific subcellular H_2_S threshold is necessary to control ATG8-dependent autophagic flux. Under cadmium-induced stress, wild-type plants presented coordinated increases in cytosolic OAS-A1 levels, H_2_S accumulation, and protein persulfidation and decreases in sulfenylation and autophagy activation. This redox reprogramming establishes a protective redox control driven by H_2_S, where cadmium-induced H_2_O_2_ promotes cysteine sulfenylation followed by persulfidation, thus preventing irreversible overoxidation. Notably, this adaptive redox control was dysregulated in all the organelle-specific mutants, regardless of changes in H_2_S or H_2_O_2_ levels, demonstrating that the entire H_2_S network is required for redox protection. Cytosolic and mitochondrial mutants presented the greatest defects in Cd-induced autophagy, indicating that these compartments play a central role in stress-adaptive recycling.

## Highlights

• H_2_S signaling in plants exerts distinct effects depending on the subcellular compartment.
• Disruption of H_2_S homeostasis triggers basal autophagy.
• All compartments are required to activate protein persulfidation under Cd.
• Cd-autophagic responses depend on the subcellular location of H_2_S.

## Graphical abstract

**Figure:**
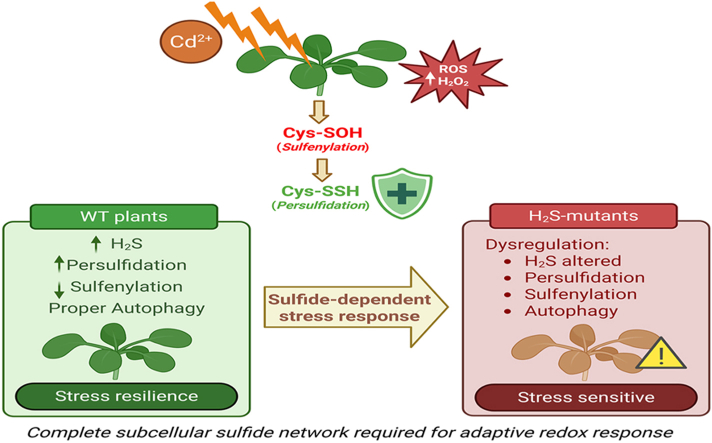

## 1 Introduction

Hydrogen sulfide (H_2_S) has traditionally been viewed as a waste byproduct or through the lens of environmental toxicology because of its characteristic pungent odor, high reactivity with metalloproteins and lethal ability to inhibit cytochrome *c* oxidase [1].

However, over the past two decades, a paradigm shift in plant biology has positioned this molecule as a crucial gasotransmitter for living organisms alongside nitric oxide (NO), carbon monoxide (CO) and hydrogen peroxide (H_2_O_2_) as fundamental regulators of cellular signaling [2,3]. H_2_S is now recognized as a master switch that governs a vast array of physiological responses. In the plant kingdom, its physiological influence is remarkably broad and includes processes that range from the initial stages of seed germination [4] to the regulation of root architecture [5] and the orchestration of stomatal dynamics [6,7]. However, H_2_S also plays important roles in resistance to pathogens [8,9] and the response to different abiotic stresses that increase plant resilience [10]. The biological versatility of H_2_S is attributed to its unique chemical properties, specifically its ability to modify proteins through persulfidation [11]. This posttranslational modification involves the conversion of a protein's cysteine thiol group (-SH) into a persulfide group (-SSH), effectively modulating the activity, stability, or localization of target proteins.

The complexity of H_2_S signaling is intrinsically linked to a multicompartmentalized enzymatic network, with specific organelle-localized production and consumption within the cell [3,10]. Unlike simple diffusion models, recent evidence suggests that plant cells maintain discrete subcellular pools of H_2_S to allow high-resolution signaling without interfering with core metabolic pathways.

In *Arabidopsis thaliana*, the primary cytosolic source of H_2_S is the enzyme l-cysteine desulfhydrase (DES1), which converts l-cysteine into sulfide and ammonia [12]. The chloroplast produces H_2_S as a central intermediate in the reductive sulfate assimilation pathway via sulfite reductase (SIR) catalysis, which reduces sulfite to sulfide as a prerequisite for cysteine synthesis [13]. The mitochondria also contribute to this “sulfide landscape” through β-cyanoalanine synthase (CAS-C1), which produces H_2_S while detoxifying cyanide [14]. Furthermore, the sulfurtransferases, including mitochondrial STR1 and cytosolic STR2, facilitate H_2_S biogenesis through the processing of 3-mercaptopyruvate [15,16]. Conversely, to prevent toxic accumulation, H_2_S levels decrease through its consumption for cysteine synthesis by O-acetylserine(thiol)lyase (OAS-TL) isoforms, OAS-A1, OAS-B, and OAS-C, which are localized in the cytosol, chloroplasts and mitochondria respectively [[17], [18], [19], [20], [21]].

One of the most critical and well-studied roles of H_2_S is its negative regulation of autophagy. Autophagy is a conserved “self-eating” mechanism essential for nutrient recycling and proteostasis maintenance [22,23]. Autophagy plays a critical role during nutrient starvation or environmental stress, by engulfing damaged organelles or protein aggregates into double-membrane vesicles called autophagosomes. The ATG8 protein family is central to this process; lipidation to generate ATG8-phosphatidylethanolamine (ATG8-PE) is important for the formation of autophagosomes [24]. Previous studies in plants have demonstrated that H_2_S acts as a constitutive repressor of autophagy. Under normal growth conditions, high H_2_S levels prevent the overactivation of autophagy, as evidenced in the *des1* mutant, which exhibits induced basal autophagy and premature senescence due to sulfide deficiency [25]. This regulation occurs via protein persulfidation, where H_2_S modifies cysteine residues in the autophagic machinery to inhibit its activity [26,27]. While the role of cytosolic H_2_S has been well documented, the specific contributions of H_2_S pools in organelles such as the chloroplast and mitochondria to the global regulation of autophagy remain poorly understood.

Furthermore, H_2_S signaling is intimately linked to the cellular level of reactive oxygen species (ROS), particularly H_2_O_2_ signaling. H_2_O_2_ accumulation leads to protein sulfenylation (Cys-SOH) [28], but if stress persists, the accumulation of ROS further irreversibly oxidizes cysteines to sulfinic (Cys-SO_2_H) or sulfonic (Cys-SO_3_H) acids. Crosstalk between H_2_O_2_ and H_2_S is mediated by the sequential oxidation of cysteine residues through ROS-induced sulfenylation, which often precedes H_2_S-mediated persulfidation. Therefore, H_2_S has been proposed to provide a protective “redox switch” by reacting with sulfenylated cysteines to form stable persulfides as a protective mechanism that preserves the proteome during stress [11,29,30].

Heavy metals such as cadmium (Cd) disrupt plant homeostasis by triggering bursts of ROS accumulation and inducing autophagy [31,32]. Plants have been shown to induce endogenous H_2_S production when subjected to Cd stress [33,34], but a clear map of how H_2_S sources mitigate oxidative damage within specific organelles or how the organelle-specific H_2_S concentration regulates the autophagic machinery is still needed.

In this work, we provide a high-resolution analysis of how localized H_2_S regulates autophagy and the “redox switch” under cadmium (Cd) stress in *Arabidopsis thaliana*. The use of a diverse library of null mutants, targeting both H_2_S production (*des1, sir, cas-c1, str1,* and *str2*) and its consumption (*oas-a1, oas-b,* and *oas-c*), helped us decipher how localized sulfide concentrations regulate both basal and stress-responsive autophagy induced by Cd. Furthermore, we investigated the molecular crosstalk between H_2_S and H_2_O_2_ by characterizing global changes in protein persulfidation and sulfenylation under Cd stress. Our findings offer a new perspective on the spatial logic of gasotransmitter signaling and its vital importance in building plant stress resilience.

## 2 Materials and methods

### 2.1 Plant material, growth conditions and treatment

The *Arabidopsis thaliana* wild-type ecotype Col-0 and *des1* (SALK_103855) [12], *oas-a1* (SALK_072213) [35], *str2* (SALK_067994) and *str1* (SALK_015593) [36], *oas-b* (SALK_021183) [37], *sir* (GABI _55A09) [13], *oas-c* (SALK_000860) [21], and *cas-c1* (SALK_022479) [38] lines, together with the double mutants *des1xst*r1 and *des1xstr2* generated by Dr. Jing Zhang (Nanjing Forestry University), were used in this work. The plants were grown in soil for twenty-five days with a photoperiod of 16 h of white light (120 μE m^−2^ s^−1^) at 20 °C and 8 h of darkness at 18 °C. For Cd treatment, plants were irrigated for 3 additional days with 200 μM CdCl_2_.

### 2.2 Analysis of chlorophyl fluorescence

Whole-rosette chlorophyll fluorescence imaging was performed using an IMAGING-PAM M-Series instrument (Walz). The maximum quantum yield of PSII (Fv/Fm) was determined after the plants were incubated in the dark for 30 min and was calculated as the ratio of the variable fluorescence (Fv) to the maximal fluorescence (Fm). Nonphotochemical quenching (NPQ) was calculated as (Fm - F'm)/F'm where the value of F'm was determined after the relaxation of NPQ for 15 min in the dark.

### 2.3 Determination of the hydrogen sulfide content

Hydrogen sulfide (H_2_S) was quantified by ultra-performance liquid chromatography–tandem mass spectrometry (UPLC–MS/MS) as previously described [39]. Arabidopsis leaves were ground to a fine powder in liquid nitrogen, and H_2_S was extracted with Tris-HCl (100 mM, pH 8.5) and derivatized with monobromobimane. Standard calibration curves were constructed with solutions of known NaHS concentrations.

### 2.4 Determination of the hydrogen peroxide content

Hydrogen peroxide (H_2_O_2_) was quantified using the Amplex Red-based method (Thermo Fisher Scientific), as described previously [40]. Arabidopsis leaves were ground to a fine powder in liquid nitrogen, and H_2_O_2_ was extracted with 50 mM sodium phosphate (pH 7.4); after centrifugation, the supernatant was used for H_2_O_2_ detection. A total of 100 μL of reaction buffer containing 25 μM Amplex Red, 10 U mL^−1^ horseradish peroxidase (HRP) and 5 μL of the supernatant in 50 mM PBS (pH 7.4) was incubated for 30 min, and changes in fluorescence caused by the formation of oxidized Amplex red (Ex/Em: 560 nm/590 nm) were recorded. Standard calibration curves were constructed with solutions of known H_2_O_2_ concentrations.

### 2.5 Determination of the Cd^2+^ content

The content of this element was determined by inductively coupled plasma–mass spectrometry (ICP–MS) using an Agilent 7800 ICP–MS instrument at the Analysis Facility of the IRNAS (Institute of Natural Resources and Agrobiology of Seville). Prior to ICP–MS analysis, fresh leaves were dried for 6 d at 65 °C. Afterward, 15 mL of a solution of 1 M HNO_3_ and 10 mM MgCl_2_ was added for 7 d of digestion at room temperature.

### 2.6 In-gel persulfidation and sulfenylation

In-gel persulfidation and sulfenylation was detected following dimedone switch detection methods as previously described [40]. For persulfidation, 150 mg of plant leaf material was ground in liquid nitrogen with 200 μL of cold PBS lysis buffer [1 × PBS (pH 7.4), 1 mM EDTA, and 2% SDS (w/v)] supplemented with 1 × protease inhibitor (Pierce™, Thermo Scientific). Afterward, the samples were incubated with 5 mM 4-chloro-7-nitrobenzofurazan (Cl–NBF) at 37 °C for 30 min in the dark. Methanol/chloroform precipitation was performed to eliminate excess Cl–NBF, and the protein pellets obtained were washed with cold methanol, dried, and redissolved in 1 × PBS with 2% (w/v) SDS supplemented with 1 × protease inhibitor. The proteins were incubated with 25 μM DAz-2/Cy-5 preclick mix at 37 °C for 30 min. Following the incubation, methanol/chloroform precipitation was performed, and the pellets were subsequently washed with methanol as described above. Protein labeling was analyzed using SDS–PAGE. After SDS–PAGE, the gels were fixed for 30 min in 12.5% methanol (v/v) and 4% acetic acid (v/v) in the dark. The gel was imaged at 640 nm to detect the Cy5 signal, which corresponds to persulfidated cysteines, and at 488 nm to detect the NBF–Cl signal, which corresponds to all cysteine residues and amino groups and served as the loading control. The level of persulfidation was quantified by calculating the Cy5/Cl–NBF fluorescence signal ratio. To quantify protein sulfenylation, the samples were labeled with DCP-Bio1 and visualized using an Alexa Fluor™ 488–streptavidin conjugate following a previously described protocol [41]. The fluorescence intensity was measured with an Ettan DIGE imager and processed with ImageJ. The Ponceau S staining intensity served as a protein loading control, and sulfenylation levels were quantified on the basis of the Alexa Fluor 488 signal intensity normalized to protein loading.

### 2.7 Real-time reverse transcription–PCR

Total RNA was extracted from Arabidopsis leaves using a Qiagen RNeasy Plant Mini Kit. RNA was reverse transcribed using oligo (dT) and the QuantiTect Reverse Transcription Kit (Qiagen) according to the manufacturer's instructions. Specific primers for each gene were designed using Vector NTI Advance 10 software (Supplementary Table S1). Real-time PCR was performed using iTaq universal SYBR qPCR Green Supermix (Bio-Rad), and the signals were detected on a CFX96 Touch Real-Time PCR Detection System (Bio-Rad) according to the manufacturer's instructions. The cycling profile consisted of 95 °C for 10 min followed by 45 cycles of 95 °C for 15 s and 60 °C for 1 min. The expression levels of the genes of interest were normalized to those of the constitutive *UBQ10* gene by subtracting the cycle threshold value of *UBQ1*0 from the cycle threshold value of the gene of interest. The results are shown as the means ±SDs from at least three independent RNA samples.

### 2.8 Immunoblotting

The plant leaf material (150 mg) was ground in liquid nitrogen with 400 mL of extraction buffer [100 mM Tris-HCl (pH 7.5), 400 mM sucrose, 1 mM EDTA, 10 mg mL^−1^ sodium deoxycholate, 0.1 mM phenylmethylsulfonyl fluoride, 10 μg mL^−1^ pepstatin A, and 4% (v/v) protease inhibitor cocktail (Roche)] and then centrifuged at 500×*g* for 10 min to obtain the supernatant fraction. The total amount of protein in the resulting supernatant was determined using a previously described method [42]. For immunoblot analyses, 30 μg of leaf protein extract was subjected to electrophoresis on acrylamide gels (Any kD Mini-protean TGX precast gels, Bio-Rad) before being transferred to nitrocellulose membranes. Anti-ATG8 (Agrisera) and secondary anti-rabbit antibodies were diluted 1:2000 and 1:30,000, respectively, in PBS containing 0.1% Tween 20 (Sigma-Aldrich) and 5% milk powder. ECL-select western blotting detection reagent (GE Healthcare) was used to detect the proteins with horseradish peroxidase-conjugated anti-rabbit secondary antibodies.

### 2.9 Statistical analysis

All the data are presented as the means of at least three independent experiments. The data were subjected to one-way or two-way analysis of variance (ANOVA, P < 0.05) using the software package for statistical analysis in GraphPad Prism 9.5.0.

## 3 Results and discussion

### 3.1 Characterization of Arabidopsis H_2_S mutants grown under physiological conditions

Studies on H_2_S signaling mechanisms have relied mainly on experimental systems in which plants are subjected to exogenous applications of a sulfide donor, such as NaHS. Alternatively, persulfidation analyses have been performed on untreated plants growing under sulfur-sufficient nutrient conditions, with the assumption that endogenous H_2_S plays a signaling role. However, H_2_S is produced endogenously by plant cells through different enzymatic reactions involved in cysteine metabolism in different cell compartments.

With the aim of determining whether different subcellular sources of H_2_S and thereby persulfidating agents could modulate H_2_S signaling in certain biological processes in a specific manner, we characterized different Arabidopsis null mutants. Given that the generation of H_2_S is closely linked to cysteine metabolism and that a balance between H_2_S and cysteine levels in each compartment is critical for adequate plant performance [43], we used plant lines in which the production of H_2_S or the H_2_S consumption through the synthesis of cysteine was affected (Table 1). The mutants expected to be defective in the enzymatic generation of H_2_S were cytosolic *des1* [12], chloroplastic *sir* [13], and mitochondrial *cas-c1* [38], and the mutants defective in the enzymatic depletion of H_2_S were cytosolic *oas-a1* [35], chloroplastic *oas-b* [37] and mitochondrial *oas-c* [21], all of which have been extensively characterized previously. We also included mutants deficient in sulfurtransferases 1 and 2, mitochondrial *str1* and cytosolic *str2*, respectively, in our analysis [36], which have been suggested to be involved in both sulfur trafficking and H_2_S biogenesis [15]. Furthermore, the corresponding double mutants that include mutation of *des1* were analyzed, the latter of which has a very well-known phenotype of reduced endogenous H_2_S levels [25].

**Table 1:** H_2_S mutants used in this work.

Mutant	Defective enzyme	Location of the enzyme	Pathway in which is involved/H_2_S outcome	References
*des1 (SALK_103855)*	L-Cysteine desulfhydrase DES1 (AT5G28030)	cytosol	cysteine degradation/H_2_S generation	[12]
*oas-a1 (SALK_072213)*	O-Acetylserine(thiol) lyase A1 (AT4G14880)	cytosol	cysteine biosynthesis/H_2_S consumption	[35]
*str2 (SALK_067994)*	Sulfurtransferase 2 (AT1G16460)	cytosol	sulfur trafficking/H_2_S generation	[36]
*oas-b (SALK_021183)*	O-Acetylserine(thiol) lyase B (AT2G43750)	chloroplast	cysteine biosynthesis/H_2_S consumption	[37]
*sir (GABI _55A09)*	Sulfite reductase (AT5G04590)	chloroplast	sulfite reduction to sulfide/H_2_S consumption	[13]
*oas-c (SALK_000860)*	O-Acetylserine(thiol) lyase C (AT3G59760)	mitochondria	cysteine biosynthesis/H_2_S consumption	[21]
*cas-c1 (SALK_022479)*	β-Cyanoalanine synthase C1 (AT3G61440)	mitochondria	cyanide detoxification/H_2_S generation	[38]
*str1 (SALK_015593)*	Sulfurtransferase 1 (AT1G79230)	mitochondria	sulfur trafficking/H_2_S generation	[36]

**Fig. 1:**
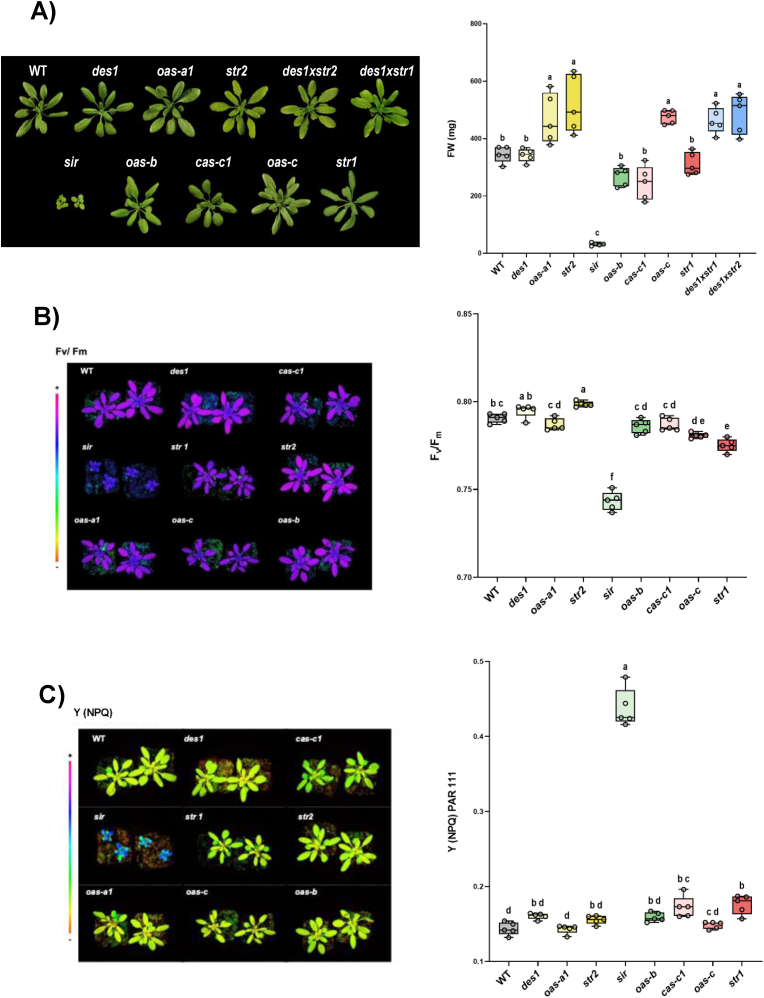
Phenotypic characterization and photosynthetic performance of the Arabidopsis H_2_S mutants. The plant lines were grown in soil under physiological conditions for 25 days. A) After this period, photographs were taken, and representative images are shown. In addition, the fresh weights of the plants were quantified. B) Representative images and quantification of the maximum PSII quantum yield (Fv/Fm) and C) of nonphotochemical quenching (NPQ) at a photosynthetic photon flux density (PPFD) of 111 μmol m^−2^ s^−1^. Color scales for each parameter are shown. Boxplots represent the distribution of biological replicates (n = 5), and individual data points are shown. Different letters indicate statistically significant differences (ANOVA, Tukey's multiple comparisons test; P < 0.05). Each mutant is represented by a different color palette as follows: mutants deficient in a cytosolic gene are yellowish, mutants deficient in a chloroplastic gene are greenish, mutants deficient in a mitochondrial gene are reddish, and double mutants are bluish.

In the initial phase, mutant plants grown in soil for 25 days under physiological conditions were screened for potential growth defects (Fig. 1A). Compared with WT plants, the most significant phenotypic differences were detected in the *sir* mutant, which were markedly smaller and exhibited a reduced fresh weight, as previously reported [13]. Furthermore, we analyzed the differences in photosynthetic performance by chlorophyll fluorescence imaging. In WT leaves, the maximum quantum yield of PSII (Fv/Fm) was close to 0.8, which was similar to that in most of the mutants, with the exception of the *sir* mutant line, the Fv/Fm of which was significantly decreased, indicating a defect in PSII (Fig. 1B). With respect to photosynthetically active radiation, the *sir* mutant presented a significantly greater NPQ value, indicating an increase in heat dissipation from PSII (Fig. 1C). These findings are consistent with those of previous studies on the characterization of two independent sir alleles that demonstrated that *sir1-1*, which was the studied in this work, is the only viable variant, leading to plants with strongly retarded growth and severe impairment of the photosynthetic sulfate reduction pathway [13].

**Fig. 2:**
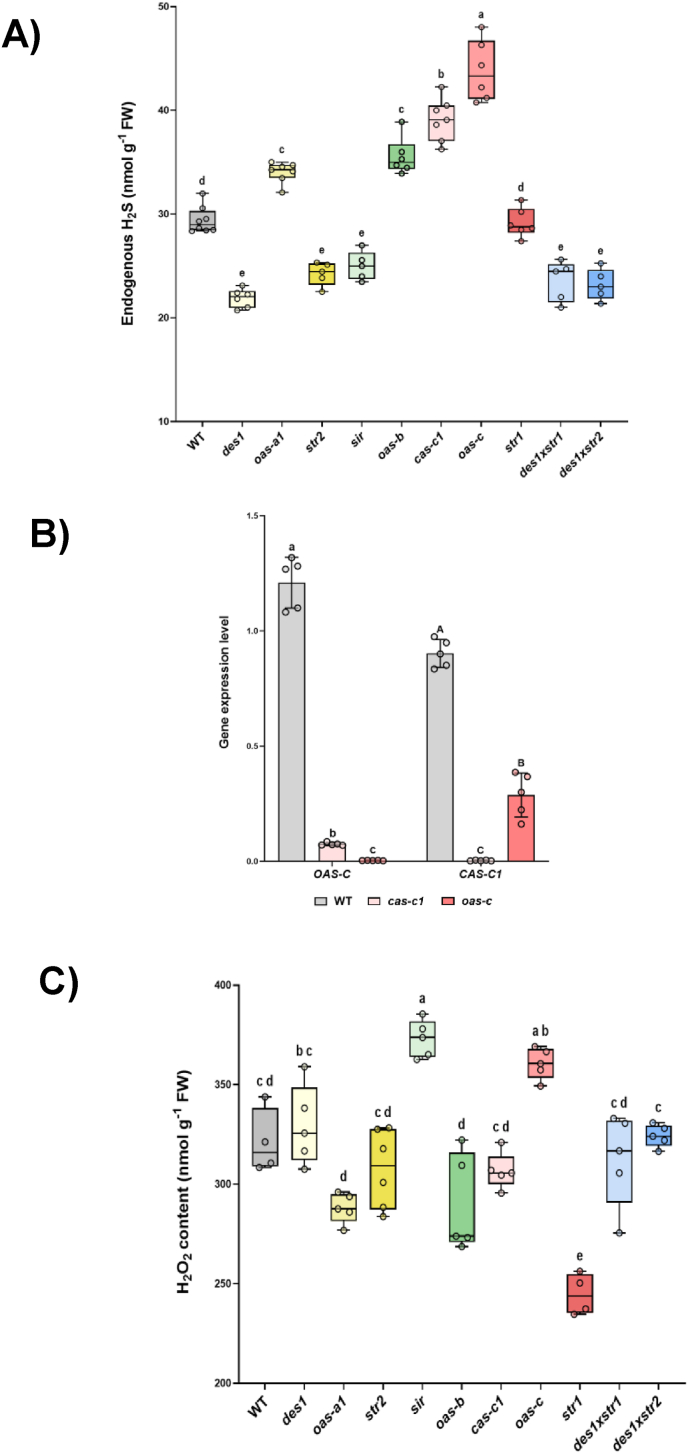
Biochemical characterization of the Arabidopsis H_2_S mutants. The plants were grown in soil under physiological conditions for 25 days, after which leaves were collected for subsequent analyses. A) Endogenous H_2_S content in the WT and mutant lines. B) Expression levels of the *OAS-C* and *CAS-C1* genes in WT, *cas-c1,* and *oas-c* plants. C) H_2_O_2_ content in the WT and mutant lines. Boxplots represent the distribution of biological replicates (n > 5), and individual data points are shown. Different letters indicate statistically significant differences (ANOVA, Tukey's multiple comparisons test; P < 0.05).

Next, we determined the endogenous H_2_S concentration in leaf extracts from the various mutants under physiological growth conditions, and found significant differences compared with that in WT plants (Fig. 2A). The *des1* mutant exhibited a reduced level of endogenous H_2_S, which is consistent with previous results [25]. As expected, other mutant lines defective in H_2_S generation, such as *sir*, *str*2*,* and the *des1xstr1* and *des1xstr2* double mutants, also presented significantly lower H_2_S levels. In contrast, *str1* displayed H_2_S concentrations comparable to those of the WT. Previous studies have suggested that STR1 and STR2 participate in H_2_S production [44]. However, on the basis of the present data, we do not find sufficient evidence to support that sulfurtransferases actively contribute to the cellular H_2_S pool, and additional experiments are needed to clarify their involvement. With respect to the level of H_2_S exhibited by the mitochondrial *cas-c1* mutant plants, we obtained unexpected results: instead of observing a reduction or no change in H_2_S content compared with that of the WT, we detected the opposite phenotype, namely, a pronounced increase in the H_2_S concentration. Additionally, compared with WT plants, mutants lacking H_2_S utilization enzymes, such as *oas-a1*, *oas-b*, and *oas-c*, presented substantial increases in H_2_S levels, which is consistent with their involvement in cysteine biosynthesis.

Previous research has demonstrated that *oas-c* and *cas-c1* mutant plants share phenotypic characteristics [21,38], and that the OAS-C and CAS-C1 enzymes function coordinately to modulate low levels of cyanide [45]. Under this premise, we hypothesized that both mutants would be indistinguishable from each other in terms of H_2_S content. Therefore, the expression levels of the *OAS-C* and *CAS-C1* genes were analyzed in both mutant lines, and a very significant decrease in the *OAS-C* expression level was detected in the *cas-c1* line, which may explain the increase in H_2_S observed in this mutant compared with the WT (Fig. 2B). This feedback loop suggests that mitochondrial H_2_S levels are governed by a delicate balance of local synthesis and consumption flux.

H_2_S-dependent protein persulfidation suggests substantial interplay between H_2_O_2_ and H_2_S signaling pathways. Protein persulfidation relies on prior protein sulfenylation because H_2_S acts as a reductant and reacts exclusively with oxidized cysteine residues [11,40]. This crosstalk is further evidenced by the overlap between ROS- and H_2_S-mediated regulation of the same biological processes, as a comparative study revealed that 82% of sulfenylated proteins in Arabidopsis are also persulfidated [6]. To delve deeper into the connection between H_2_O_2_ and H_2_S, we quantified the H_2_O_2_ content within the various H_2_S mutants (Fig. 2C).

When we compared the patterns in H_2_O_2_ levels across the mutant lines, most displayed profiles similar to those observed in WT plants. These plants included mutants with impaired H_2_S production, such as the cytosolic *des1* and *str2* lines, the mitochondrial *cas-c1* line, and the double mutants. In addition, mutants defective in H_2_S consuming enzymes, such as the *oas-a1* and *oas-b* lines, also exhibited H_2_O_2_ levels comparable to those of the WT. In contrast, among the H_2_S production-impared mutants, the chloroplastic *sir* line showed a clear increase in H_2_O_2_ levels. This pattern reflects the essential role of SIR and shows that its downregulation triggers severe perturbations, including oxidative stress. These results are consistent with previously reported strong disruptions in primary and secondary metabolism [13], where an increase in steady-state cysteine levels was reported in the *sir* mutant, which was proposed to contribute to the maintenance of steady-state GSH levels. Therefore, the fluctuations in H_2_O_2_ levels demonstrate the metabolic complexity of the *sir* mutant. Similarly, the H_2_S consumption-defective mitochondrial *oas-c* mutant exhibited a substantial increase in H_2_O_2_ levels. In contrast, the H_2_S production-defective mitochondrial *str1* mutant showed much lower H_2_O_2_ levels than in the WT. These results suggest that the loss of the sulfurtransferase STR1 may reduce mitochondrial ROS production or instead induce a compensatory increase in the efficiency of ROS scavenging, two very intriguing possibilities that require further research. Overall, we conclude that H_2_O_2_ accumulation is not strictly dictated by the subcellular compartment in which H_2_S metabolism is disrupted. Moreover, considering the high mobility of H_2_O_2_ within cells, these measurments from total extracts likely reflect a complex and integrated cellular response rather than an isolated subcellular compartimentalization effect.

### 3.2 Autophagic activity of Arabidopsis H_2_S mutants grown under physiological conditions

H_2_S regulates autophagy in both animal and plant systems [46]. Indeed, the initial indication of this regulation emerged from the characterization of the *des1* mutant, which exhibited increased accumulation and lipidation of ATG8 proteins. These observations suggest the induction of autophagy in a mutant with reduced levels of H_2_S. Genetic complementation or exogenous H_2_S application restored the induced-autophagy phenotype [25]. To assess whether the mode of autophagy regulation by H_2_S is subcellular compartmental specific, we analyzed the transcriptional levels of the nine different Arabidopsis *ATG8* genes and the accumulation and lipidation of ATG8 proteins in all H_2_S mutants.

**Fig. 3:**
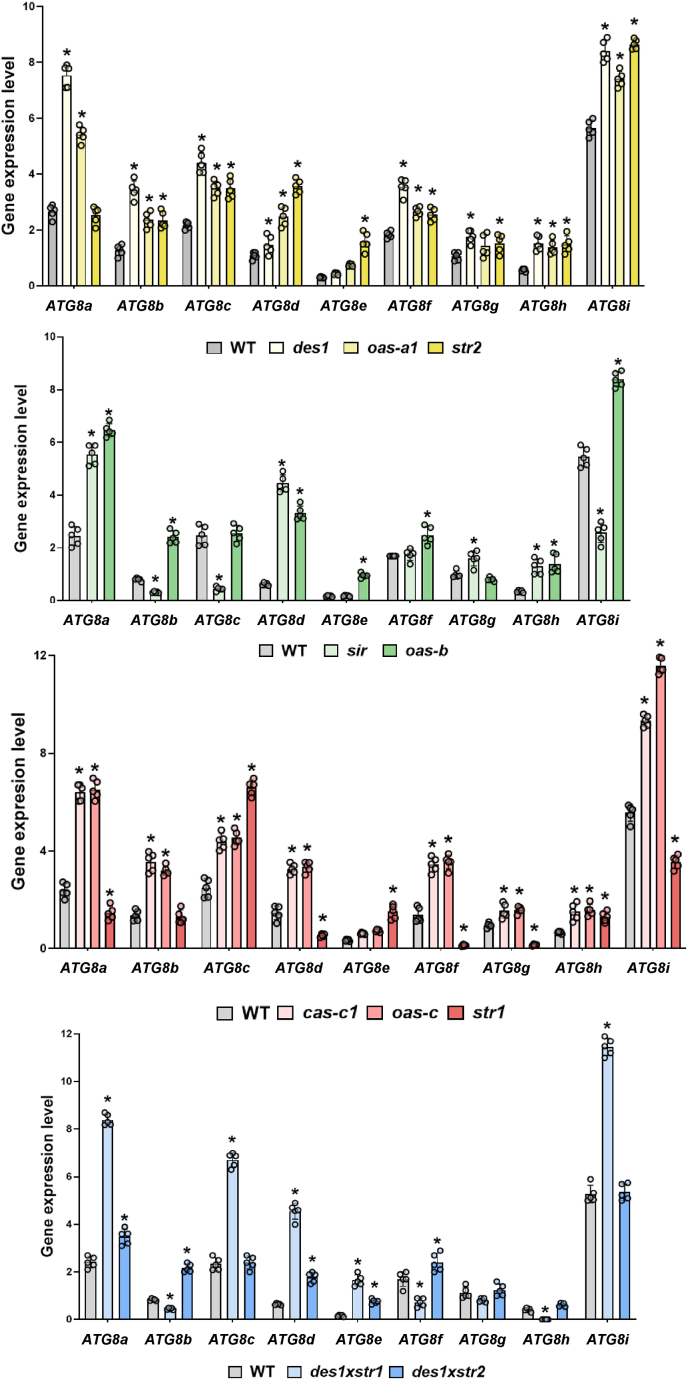
Expression levels of the *ATG8* gene family in Arabidopsis WT and the H_2_S mutants. Real-time RT–PCR analysis of the nine Arabidopsis ATG8 genes was performed on the indicated plant samples. The values are presented as the means ±SDs (n > 5). Asterisks indicate statistically significant differences compared with the WT (two-way ANOVA, Tukey's multiple comparisons test; P < 0.05). The data are presented in distinct panels to improve comprehension on the basis of the color distribution indicated in Fig. 1.

WT plants presented differential levels of all *ATG8* transcripts, which were significantly increased in the majority of the H_2_S mutants, regardless of the subcellular location or the nature of the mutant, i.e., H_2_S deficient or H_2_S accumulator (Fig. 3). This suggests that any significant deviation in sulfide concentration, whether it is a deficiency or an excess, is sensed by the cell as a metabolic stress that triggers the autophagic machinery. Such an effect may imply that the maintenance of a specific H_2_S threshold is required to prevent the activation of the core autophagic machinery; however, given the current lack of organelle-specific H_2_S quantification tools, this hypothesis requires further experimental validation. In line with this view, previous findings in animal models have shown that both H_2_S deficiency and excessive H_2_S exposure disrupt proteostasis through distinct but overlapping autophagic pathways [47].

Some exceptions to the overall increase in *ATG8* transcript levels were observed. Specifically, the *sir* mutant presented reduced levels of *ATG8b, c,* and *i* transcripts; the *str1* mutant exhibited decreased levels of *ATG8a, d, f, g,* and *i* transcripts; and the *des1xstr1* double mutant presented reduced expression of *ATG8b, f,* and *h*. These distinct transcriptional signatures observed in certain defective lines may be associated with alterations in H_2_S metabolism at the organelle level. In this context, previous studies on mitochondrial retrograde signaling have shown that severe organelle dysfunction can lead to the specific suppression of certain core autophagy genes to prevent the premature clearance of partially functional organelles. This survival strategy has been well documented in yeast and recently suggested in plants [48].

**Fig. 4:**
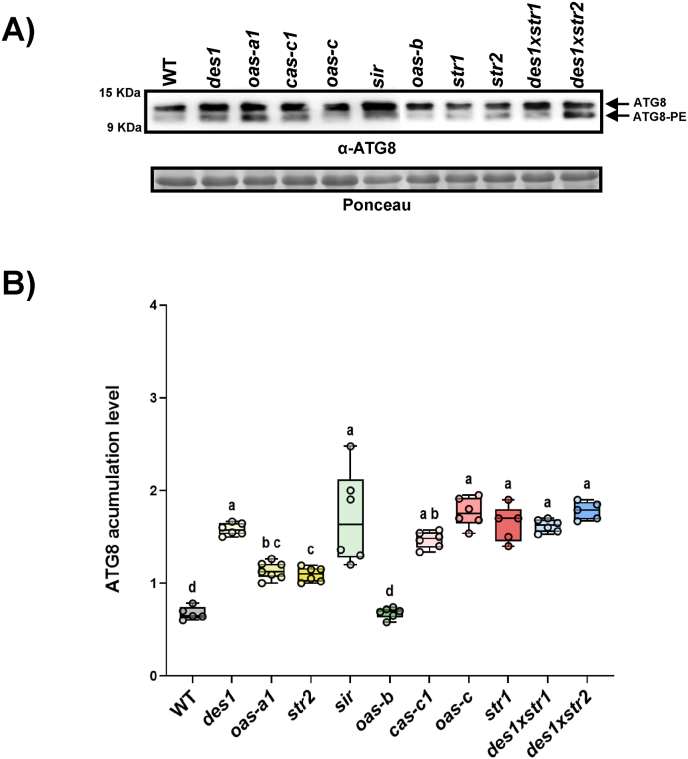
Immunoblot analysis of endogenous ATG8 proteins in Arabidopsis WT and the H_2_S mutants. A) Total protein extracts were subjected to SDS–PAGE and immunoblot analysis with an anti-ATG8 antibody. The positions of the nonlipidated ATG8 form and the lipidated ATG8–PE form are indicated. Ponceau staining was used as a protein loading control. A representative image is shown. B) Quantification of ATG8 levels in the WT and mutant lines. The relative band intensities were normalized to those of the protein loading control. Boxplots represent the distribution of biological replicates (n ≥ 5), and individual data points are shown. Different letters indicate statistically significant differences (ANOVA, Tukey's multiple comparisons test; P < 0.05).

At the protein level, the immunodetection of ATG8 proteins, both unmodified and lipidated, showed significantly greater ATG8 accumulation in most mutant lines than in the WT line, which is consistent with the observed increase in transcription levels (Fig. 4). Notably, the chloroplastic mutants exhibited distinct patterns. Compared with the WT, the H_2_S-deficient *sir* mutant exhibited greater ATG8 accumulation, whereas the H_2_S-accumulator *oas-b* mutant displayed ATG8 levels comparable to those observed in the WT. This discrepancy suggests that chloroplastic regulation of autophagy may occur at posttranscriptional level or through changes in protein stability, resulting in increased sensitivy to H_2_S scarcity rather than excess, and this phenomenon is likely related to the amount of H_2_O_2_ accumulated in each mutant.

Collectively, these findings indicate that genetic perturbations affecting H_2_S homeostasis, regardless of their subcellular location, trigger the activation of autophagy. These observations suggest that H_2_S may modulate an upstream component of the autophagy regulatory network. Notably, several persulfidated proteins implicated in autophagy regulation have been identified, including the serine/threonine kinase TOR and its effector proteins RAPTOR 1 and LST8 [49,50], thereby supporting the conclusion of general autophagy regulation by H_2_S. Similarly, the sulfur limitation-induced downregulation of TOR in shoots and the consequent activation of autophagy have been previously demonstrated [51].

### 3.3 Analysis of plant responses to heavy metal stress in H_2_S mutant lines

In recent years, increasing attention has been given to the role of H_2_S in regulating plant responses to various abiotic stresses and promoting plant resilience [10]. To investigate whether distinct subcellular locations in which H_2_S is generated could be specifically activated under specific stress conditions, we initially focused on heavy metal stress, particularly Cd stress. Several studies have previously demonstrated that Cd exposure induces H_2_S generation, while other reports have shown that exogenous supplementation with H_2_S increases Cd tolerance, mainly through increased antioxidant defenses and altered Cd accumulation [52,53].

We first studied the kinetics of Cd treatment on 25-day-old water-irrigated WT plants (control) that were treated with 200 μM CdCl_2_ by collecting leaf samples at 24 h and 3 and 6 d for subsequent analysis (Fig. S1). The H_2_O_2_ content and autophagic ATG8 accumulation, both of which are well known to be induced in response to Cd treatment, were subsequently quantified [31,54,55], and the results revealed the highest level of induction after 3 days of treatment. In addition, the quantification of Cd revealed significant accumulation in leaves at this time point. This temporal pattern suggests that the 3-day window represents a state of established physiological stress where metabolic redirection toward defense mechanisms is maximized. Therefore, this time was selected for the following studies.

**Fig. 5:**
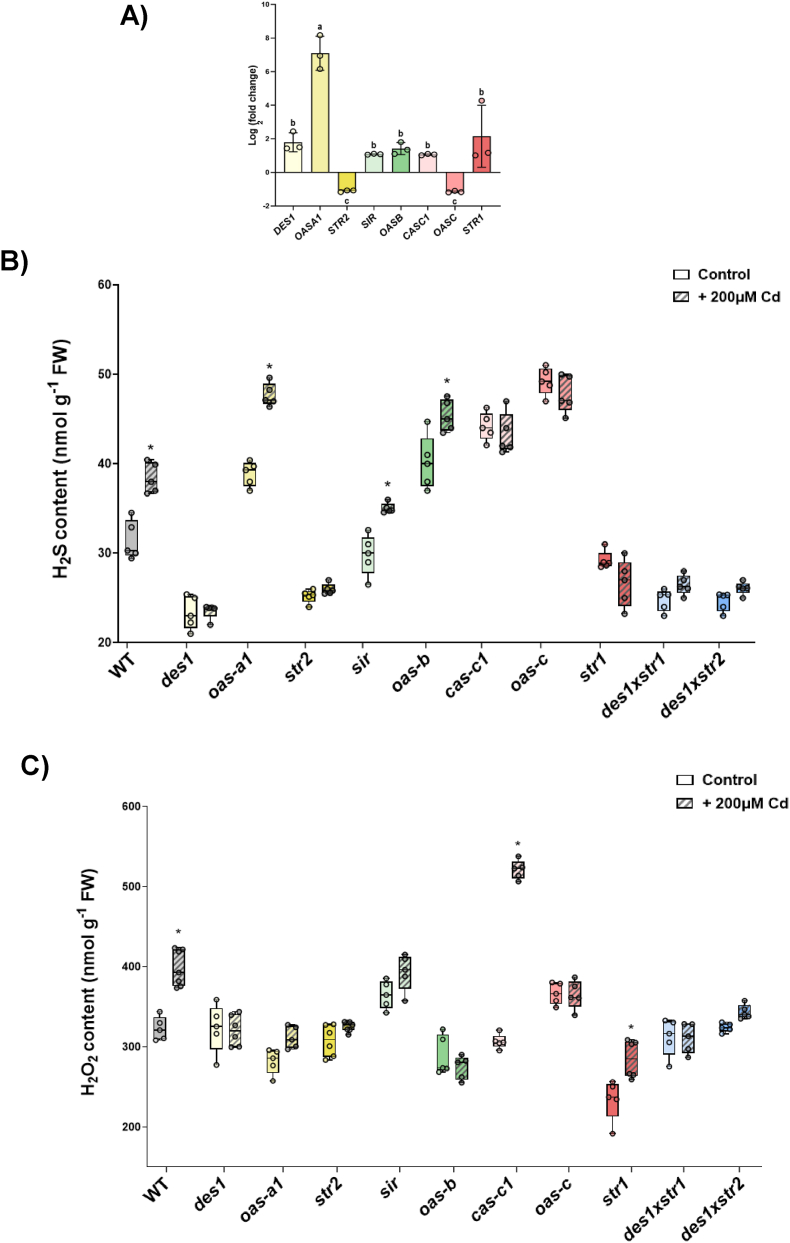
Effects of Cd treatment on Arabidopsis plant lines. A) Relative expression levels of genes involved in H_2_S metabolism in WT plants treated with 200 μM CdCl_2_ for 3 d. Real-time RT–PCR analysis of the expression of the indicated genes in control (no treatment) and Cd-treated WT plants. The transcript levels were normalized to those of the constitutive *UBQ10* gene. The values are presented as the means ±SDs (n = 3) and represent the transcript levels of each gene in Cd-treated plants relative to those in control plants. Different letters indicate statistically significant differences (ANOVA, Tukey's multiple comparisons test; P < 0.05). B) Endogenous H_2_S content in WT and mutant lines under control conditions and after 3 d of treatment with 200 μM CdCl_2_. Boxplots represent the distribution of biological replicates (n = 5), and individual data points are shown. Asterisks indicate statistically significant differences between Cd-treated and control plants within each genotype (ANOVA, Šídák's multiple comparisons test; P < 0.05). C) H_2_O_2_ content in WT and mutant lines under control conditions and after 3 d of treatment with 200 μM CdCl_2_. Boxplots represent the distribution of biological replicates (n = 5), and individual data points are shown. Asterisks indicate statistically significant differences between Cd-treated (hatched boxes) and untreated plants (open boxes) within each genotype (ANOVA, Šídák's multiple comparisons test; P < 0.05).

To investigate the impact of Cd treatment in mutant plants affected in H_2_S metabolism, we first analyzed the expression levels of related genes in WT plants. The expression of most of genes analyzed did not significantly change after 3 d of Cd treatment, with the exception of the *OAS-A1* gene, whose expression significantly increased. Slight decreases in the expression levels of the *STR2* and *OAS-C* genes were also detected (Fig. 5A). These results highlight *OAS-A1* as the main gene regulated by Cd, representing the predominant plant response to increased cysteine biosynthesis, a process essential for the synthesis of phytochelatins (PCs) involved in Cd tolerance, as previously described [56]. Although OAS-A1 is the most abundant OASTL isoform [17,19,35], its overexpression in Arabidopsis increases plant tolerance to heavy metal stress, indicating that the availability of cytosolic cysteine may constitute a limiting step in phytochelatin biosynthesis [57]. Therefore, the specific induction of the cytosolic OAS-A1 isoform, while organelle-specific isoforms such as OAS-C are slightly repressed, suggests strategic prioritization of cytosolic cysteine production to satisfy the high demand for PCs and glutathione (GSH) required for immediate metal chelation in the cytosol prior to vacuolar sequestration [58].

We subsequently evaluated the effects of Cd treatment on WT plants and the different H_2_S mutants. Quantification of endogenous H_2_S levels revealed a substantial increase in Cd-treated WT plants compared with those in untreated plants (Fig. 5B), which may reflect an increased demand for H_2_S to sustain increased cysteine biosynthesis, as previously discussed. In contrast, Cd treatment did not significantly alter H_2_S levels in most of the H_2_S mutants. However, mutants impaired in cytosolic (*oas-a1*) and chloroplastic (*oas-b*) cysteine biosynthesis exhibited a marked increase in H_2_S content under Cd exposure. In these mutants, the H_2_S production machinery (DES1 in the cytosol and SIR in the chloroplast) remains fully active, whereas enzymes involved in H_2_S utilization are absent, resulting in significant H_2_S accumulation. This metabolic bottleneck highlights that cellular H_2_S levels rely on a fine balance between H_2_S production and consumption and supports the notion that both the cytosol and chloroplasts are active sites of H_2_S synthesis. Additionally, an increase in the H_2_S level was detected in the *sir* mutant after Cd treatment, which may similarly reflect the need for more H_2_S to support cysteine biosynthesis for metal chelation.

We further examined the effect of Cd exposure on H_2_O_2_ accumulation given that Cd induces ROS production and that functional interplay between H_2_S and H_2_O_2_ signaling pathways has been previously reported. As expected, Cd treatment resulted in a significant increase in H_2_O_2_ content in WT plants (Fig. 5C). However, the *des1* and *str2* mutants were significantly impaired in triggering such ROS induction under Cd exposure. These findings suggest that when H_2_S is not produced in the cytosol, plants are unable to respond to metal stress through the ROS-mediated signaling pathway. Deficiency of *DES1* results in an increase in the total cysteine content, and consequently, the mutant plants exhibit increased antioxidant defenses and greater tolerance to conditions that induce oxidative stress, including Cd stress [12]. This finding is also supported by recent literature redefined H_2_S as a facilitator of ROS signaling rather than just a damper. For instance, H_2_S produced by *DES1* can posttranslationally modify the respiratory burst oxidase homolog D (RBOHD) through persulfidation in guard cells [59]. If *DES1* is missing, the activation of RBOHD is compromised, leading to the impaired ROS production that we observed in the *des1*, *str2* and respective double-mutant line. This impairment likely explains the hypersensitivity of these mutants, since they fail to respond with an effective defense because they cannot initiate the necessary oxidative signal.

In contrast, alterations in H_2_O_2_ levels were detected only in mutants deficient in mitochondrial proteins. Specifically, the *str1* mutant exhibited a smaller increase, whereas the *cas-c1* mutant displayed pronounced H_2_O_2_ accumulation under Cd exposure. This phenotype in *cas-c1* may be associated with elevated levels of cyanide [38], a potent inhibitor of the mitochondrial respiratory chain, which can exacerbate mitochondrial ROS production. The data indicate that in the mitochondrial mutant *cas-c1*, metabolic stress and plant fitness do not change in parallel, suggesting that these traits can be uncoupled. As discussed above, the loss of *CAS-C1* causes cyanide to accumulated inside the cell [14], and cyanide strongly inhibits cytochrome *c* oxidase of the mitochondrial electron transport chain. With the additional stress from Cd treatment, this inhibition likely increases electron leakage from the electron transport chain, which is consistent with the sharp increase in ROS levels measured in our biochemical assays. In contrast, our phenotypic data indicated that *cas-c1* remained stable (Fig. 1A), and that its fresh weight, Fv/Fm, and Y(NPQ) did not differ from those of the WT. This suggests that, in this mutant, the induction of ROS reflects a local metabolic byproduct of mitochondrial dysfunction, not a broad signal of cellular death. These plants may rely on other compensatory processes, such as induction of the alternative oxidase (AOX) pathway, as previously shown [38], to route electrons around inhibited electron transport chain complexes and sustain photosynthetic performance despite the mitochondrial ROS burden.

Overall, in our study, the levels of H_2_S and H_2_O_2_ under control conditions and following Cd treatment correlated with subcellular localization on the basis of the specific H_2_S mutants analyzed. Quantitative assessment of these metabolites via subcellular microscopy imaging using fluorescent sensors offers a highly precise analytical approach, as it allows for the detection of molecules within specific organelles. While such high-resolution quantification is currently feasible for H_2_O_2_, an equivalent fluorescent sensor enabling the organelle-specific quantification of H_2_S in plant cells has yet to be reported. The development of such tools would undoubtedly refine our spatial understanding of signaling crosstalk beyond the scope of currently available methodologies.

### 3.4 Evaluation of the crosstalk between protein persulfidation and sulfenylation in H_2_S mutants under cadmium stress

H_2_S signaling occurs through protein persulfidation, a process in which H_2_S reacts with oxidized cysteine residues in proteins, primarily sulfenylated residues. This mechanism implies that there is crosstalk between the H_2_O_2_ and H_2_S signaling pathways, where protein persulfidation relies on prior protein sulfenylation [11]. To assess the impact of Cd stress on this crosstalk, we analyzed protein persulfidation and sulfenylation levels in the different H_2_S mutants under Cd exposure. Notably, the data presented here are global persulfidation and sulfenylation levels; therefore, future proteomic analyses are needed to identify specific protein targets of interest.

**Fig. 6:**
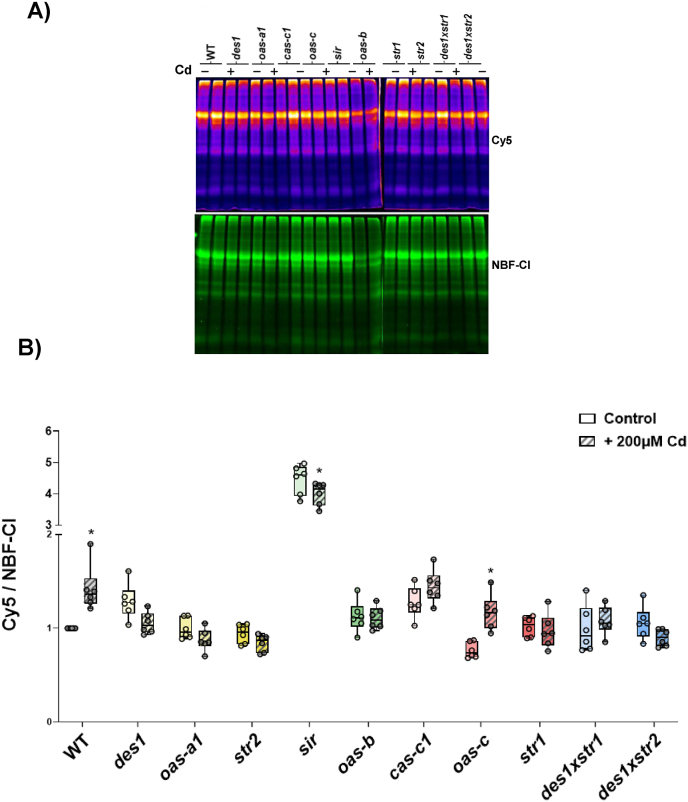
Effects of Cd treatment on total persulfidation levels in Arabidopsis WT and the H_2_S mutants. WT and H_2_S mutant plants were left untreated or treated with 200 μM CdCl_2_ for 3 d, and the protein extracts obtained were subjected to in-gel detection of persulfidation using the dimedone switch method. Visualization of the Су5 signal is shown in fire pseudocolor. Green corresponds to the total protein load (NBF–Cl). A) Representative images of the gels are shown. B) Quantification of the ratio of the Cy5/NBF–Cl signals. Boxplots represent the distribution of biological replicates (n = 6), and individual data points are shown. Asterisks indicate statistically significant differences between Cd-treated (hatched boxes) and untreated plants (open boxes) within each genotype (ANOVA, Šídák's multiple comparisons test; P < 0.05).

Prior to investigating the impact of Cd on this crosstalk in different H_2_S-related genotypes, the level of protein persulfidation in WT and mutant plants grown under physiological conditions was analyzed (Fig. S2A). Overall, compared with the WT, no substantial differences were detected in total protein persulfidation among the various mutants, except for the *sir* mutant, which exhibited a pronounced increase in persulfidation. Additionally, compared with the WT, the *des1* and *cas-c1* mutants displayed slightly higher persulfidation levels, whereas the *oas-c* mutant presented slightly lower levels. On the basis of these data, no apparent correlation between persulfidation and H_2_S content (Fig. 2A) could be established. After exposure to Cd stress (Fig. 6), a marked increase in protein persulfidation was subsequently detected in the WT plants. In contrast, the persulfidation levels of the mutant lines remained largely comparable to those observed under nonstress conditions. The only exceptions were the *sir* mutant, which exhibited a slight decrease in persulfidation, and the *oas-c* mutant, which showed a modest increase. Furthermore, analysis of the effect of Cd exposure on the H_2_S content in each line (Fig. 5B) revealed a correlation in the WT only, where Cd treatment induced an increase in H_2_S levels, thereby promoting protein persulfidation. In contrast, the *oas-a1* and *oas-b* mutants, which exhibited an increase in H_2_S accumulation in response to Cd, did not show altered persulfidation levels, whereas *sir,* in which the H_2_S content also increased, showed a slight reduction in persulfidation levels. Moreover, the *oas-c* mutant displayed increased persulfidation levels, although this effect did not correlate with its H_2_S content, which decreased slightly but not significantly.

Analysis of total protein sulfenylation levels under control conditions revealed greater variability among the different H_2_S mutants compared with the WT than was observed for protein persulfidation (Fig. S2B). Significant increases in sulfenylation levels were detected in the cytosolic *des1* and *oas-a1* mutants, as well as in the chloroplastic *oas-b* mutant. In contrast, the mitochondrial *cas-c1, str1,* and *oas-c* mutants exhibited significantly lower sulfenylation levels under physiological conditions, with the *oas-c* mutant showing a pronounced decrease. No clear correlation with the H_2_O_2_ content measured in the different mutant lines (Fig. 2C) could be established, with the exception of the *oas-c* mutant, which displayed an inverse relationship characterized by the lowest sulfenylation level and the highest H_2_O_2_ content.

**Fig. 7:**
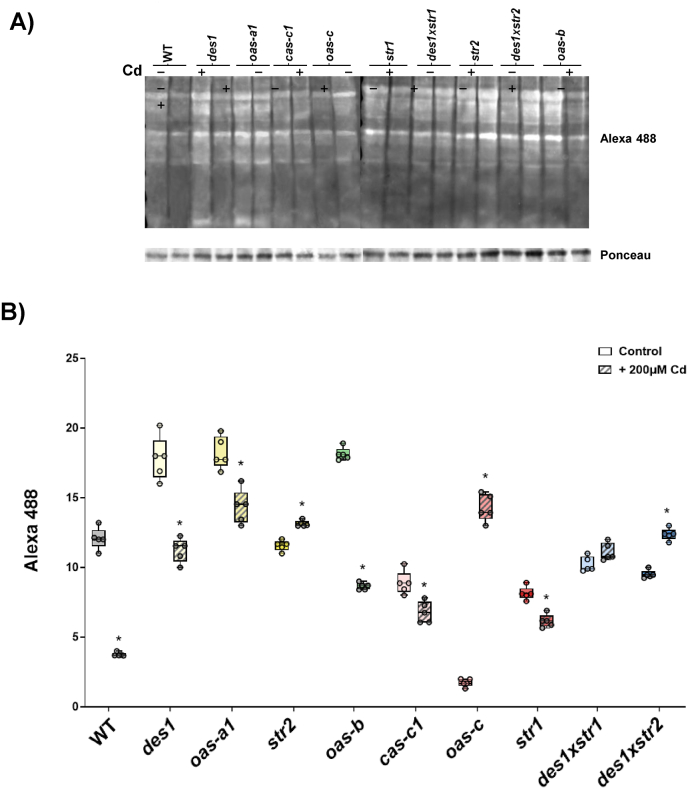
Effects of Cd treatment on total sulfenylation levels in Arabidopsis WT and the H_2_S mutants. WT and H_2_S mutant plants were left untreated or treated with 200 μM CdCl_2_ for 3 d, and the protein extracts obtained were subjected to in-gel detection of protein sulfenylation levels by labeling with DCP-Bio1 and visualized with Alexa Fluor™ 488-conjugated streptavidin. Ponceau staining was used as a protein loading control. A) A representative image is shown. B) Quantification of Alexa 488 signal intensities normalized to the protein loading control. Boxplots represent the distribution of biological replicates (n = 5), and individual data points are shown. Asterisks indicate statistically significant differences between Cd-treated (hatched boxes) and untreated plants (open boxes) within each genotype (ANOVA, Šídák's multiple comparisons test; P < 0.05).

Further analysis of the effects of Cd on sulfenylation levels (Fig. 7), showed that WT plants exhibited a pronounced decrease in protein sulfenylation, which contrasts with the opposite trend observed for persulfidation. Furthermore, unlike the uniform response detected for persulfidation, Cd treatment had different effects on sulfenylation across all the mutant lines. Specifically, upon Cd treatment, the sulfenylation levels of the mutants *des1, oas-a1, oas-b, cas-c1,* and *str1* decreased, whereas those of the mutants *str2, oas-c*, and the double mutant *des1xstr2* increased. When the effect of Cd exposure on the H_2_O_2_ content was analyzed (Fig. 5C), a negative correlation was evident in WT plants and the *cas-1* and *str1* mutant lines, and, to a lesser extent, in *oas-a1*. These genotypes exhibited Cd-induced H_2_O_2_ accumulation accompanied by a substantial reduction in sulfenylation levles. In contrast, the mutants *des1, str2, oas-b,* and *oas-c* displayed increased sulfenylation levels together with reduced or unchanged H_2_O_2_ concentrations.

Among cellular organelles, mitochondria are particularly sensitive to cadmium toxicity. Cd^2+^ ions disrupt the mitochondrial electron transport chain (ETC), altering the redox environment and promoting the accumulation of ROS. To maintain electron flow through the TCA cycle while limiting excessive ROS production, the alternative oxidase pathway is activated [52,54]. In *cas-c1*, although the level of ROS was high because of cyanide-mediated inhibition of the ETC [38] and increased further upon Cd exposure, the plant might still have enough localized H_2_S, detected even in the absence of Cd, to maintain basal persulfidation of essential mitochondrial enzymes. In contrast, the *oas-*c mutant displayed a distinct response, exhibiting higher sulfenylation levels following Cd treatment, which was attributed to the loss of persulfidation-dependent protection. These findings indicate that the physiological response of *cas-c1* is intrinsically conditioned by its elevated cyanide accumulation phenotype relative to that of *oas-c*, resulting in a distinct oxidative and redox behavior compared with that of the *oas-c* mutant [21,38]. Moreover, chloroplasts are a substantial source of ROS under Cd exposure, as Cd disrupts multiple aspects of photosynthesis, including the photosynthetic electron transport chain [52,54]. Interestingly, the *oas-b* mutant exhibited markedly lower levels of sulfenylation and a reduced H_2_O_2_ content. These phenomena may protect chloroplastic proteins from overoxidation via protein persulfidation [11].

Overall, these findings indicate that the impact of Cd exposure on the crosstalk between H_2_O_2_ and H_2_S signaling pathways occurs exclusively in WT plants. Cd treatment promotes ROS accumulation in WT plants, which may lead to cysteine overoxidation and subsequent protein degradation. To counteract this effect, WT plants exhibit a specific adaptative response involving the induction of H_2_S, which reacts with sulfenylated cysteines, thereby reducing sulfenylation levels while increasing persulfidation, as observed in WT plants. This transient protective mechanism has previously been reported in different biological systems [40,41]. Moreover, this response appears to require the complete set of subcellular components involved in H_2_S biosynthesis, as none of the mutants analyzed in this study showed a significant increase in protein persulfidation in response to Cd stress.

### 3.5 Regulation of autophagy in H_2_S mutants under cadmium stress

**Fig. 8:**
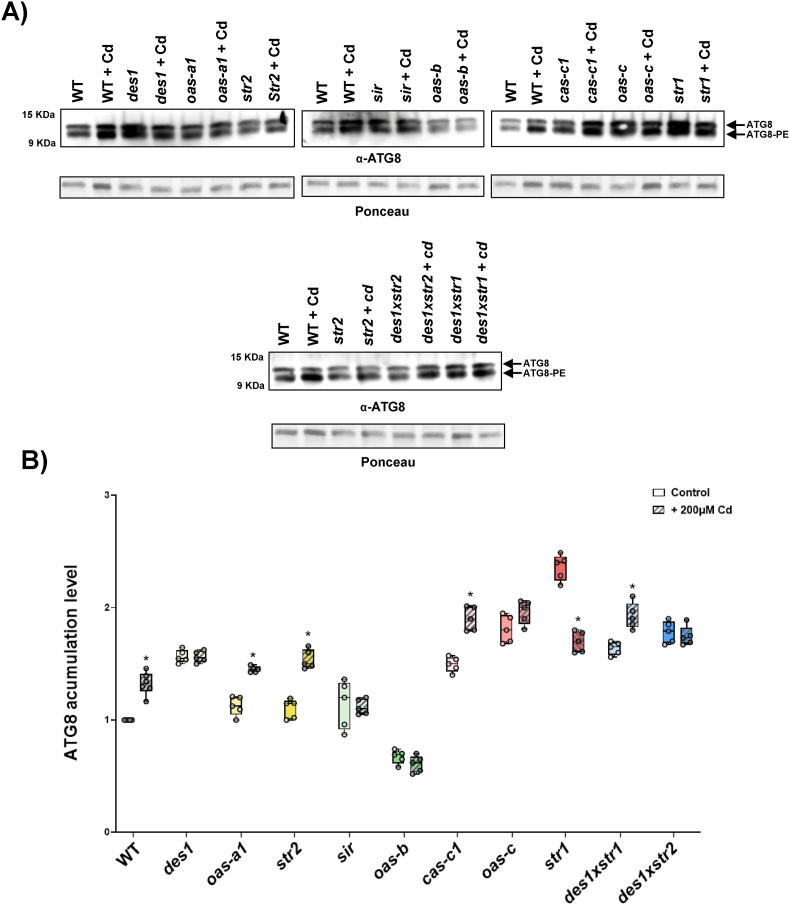
Effects of Cd treatment on ATG8 accumulation in Arabidopsis WT and the H_2_S mutants. A) Protein extracts from WT and the indicated mutant lines, either untreated or treated with 200 μM CdCl_2_ for 3 d, were analyzed by immunoblotting using an anti-ATG8 antibody. The positions of the nonlipidated ATG8 form and the lipidated ATG8–PE form are indicated. Ponceau staining is shown as a loading control. Representative images are shown. (B) Quantification of ATG8 levels for each genotype under control or Cd-treated conditions. Boxplots show the distribution of biological replicates (n = 5), and individual data points are shown. Asterisks indicate statistically significant differences between Cd-treated (hatched boxes) and untreated plants (open boxes) within each genotype (ANOVA, Šídák's multiple comparisons test;P < 0.05).

The induction of autophagy under stress conditions, as well as the negative regulation of plant autophagy by H_2_S, have been well established [26]. In particular, Cd exposure has been shown to induce autophagy [31,55]; however, no information is currently available regarding autophagy regulation by H_2_S. Therefore, we examined the autophagic response to Cd treatment in different H_2_S mutants by immunodetection of the ATG8 protein forms (Fig. 8). Because of the large number of protein samples to be analyzed, several independent immunoblotting experiments were performed. In all cases, samples from the untreated and Cd-treated WT plants were included for comparison. Both ATG8 and its lipidated form were detected in all the plant lines, and total ATG8 accumulation was quantified under untreated and Cd-treated conditions for each genotype. A significant increase in ATG8 accumulation was detected in WT plants under Cd exposure, which is in agreement with previous reports [32,60]. Interestingly, the autophagic responses to Cd followed a pattern that depended on the subcellular compartment in which the defective protein was located rather than on the specific type of mutation (whether it affected H_2_S generation or H_2_S consumption).

The cytosolic responses were very intriguing. In contrast to *oas-a1* and *str2*, which showed an autophagy induction similar to that of the WT, the *des1* mutant showed no response upon exposure to cadmium. This limited sensitivity may reflect that, in *des1*, the autophagy system is already operating near its maximum capacity. Because the H_2_S-mediated inhibitory signal that normally suppresses this pathway is absent or limited, the pathway remains active under control conditions (Fig. 4) even when there is no external stress, as previously suggested [61,62]. Cd treatment is unable to induce autophagy in *des1* plants because the pathway is already overinduced.

The mitochondrial mutants exhibited a more complex pattern. In *cas-c1*, ATG8 expression clearly increased, which likely reflects the large ROS burst detected (Fig. 5C). This acute increase in oxidative activity likely serves as the main signal, outweighing any inhibitory mechanisms that are already in place. In contrast, the results with the *str1* mutant was unexpected: ATG8 levels decreased after cadmium treatment, making it the only mutant line that displayed this trend; thus, further investigation is warranted to elucidate the underlying mechanism. While the mechanisms underlying this response remain to be fully elucidated, these findings indicate that compared with the other mutants, the *str1* background maintains a different autophagic status under these specific conditions. This decrease in ATG8 accumulation suggests that the mitochondrial dysfunction characteristic of *str1* may lead to a distinct regulatory outcome when these plants are subjected to the proteotoxic load of cadmium at this concentration and for this exposure duration.

The chloroplast-localized mutants, including *sir* and *oas-b*, seemed to be largely unaffected by Cd, since their ATG8 levels remained mostly unchanged. These findings indicate that chloroplasts are unlikely to act as the main site of signal control for heavy metal-induced autophagy; instead, the key regulatory activity appears to occur in the cytosol and mitochondria.

Taken together, these data support the conclusion that H_2_S functions as a central regulator of cellular activity. It determines whether cadmium stress triggers an adaptive autophagy response that supports cell survival or instead leads to the loss of homeostasis.

In WT plants, a timely burst of H_2_S counteracts the increase in ROS resulting from cadmium treatment, allowing the cell to recover and remove damaged proteins. Mutants unable to maintain this balance lose the ability to coordinate this protective process.

The finding that *des1* does not respond to Cd-triggered autophagy supports the view that cytosolic H_2_S is the main signal that resets the timing of autophagy [46]. The increased basal autophagy observed in mitochondrial mutants suggests that organelle-specific H_2_S pools are needed to prevent the early activation of recycling pathways such as autophagy. Therefore, autophagic responses are predominantly influenced by perturbations in the cytosol and mitochondria. Moreover, as previously proposed, the response of plants to heavy metal stress depends largely on their redox state [32,63], and H_2_S might act as a main regulator of that state.

## 4 Conclusion

Our results indicate that H_2_S signaling in plants occurs mainly at specific subcellular sites and is shaped by organelle-based hubs. The chloroplast functions as a primary hub required for basal development, as impaired sulfite reduction severely compromises growth and photosynthetic performance. In contrast, mitochondrial H_2_S homeostasis relies on a precise balance between its synthesis and use and is integrated with the cyanide detoxification pathway. Under physiological conditions, any genetic imbalance in H_2_S homeostasis, whether deficiency or accumulation, can trigger basal autophagy. These results support the view that H_2_S functions as a finely tuned repressor of autophagy progression, potentially modulating an upstream regulatory component. Under cadmium stress, the coordinated adaptive response of wild-type plants is characterized by the induction of the cytosolic gene *OAS-A1*, which is involved in promoting cysteine and phytochelatin biosynthesis. This response is accompanied by elevated endogenous H_2_S levels, which confer protection through protein persulfidation, thereby preventing ROS-mediated overoxidation and degradation. We present evidence of a cellular redox wave during cadmium stress in which H_2_S shifts harmful protein sulfenylation toward persulfidation, which appears to be protective. Because this switch failed in every organelle-specific mutant we tested, we infer that proteome stability depends on a fully functional subcellular sulfide network. Moreover, the autophagic responses to Cd followed a pattern determined by the subcellular compartment in which H_2_S homeostasis was disrupted and is independent of whether the mutation impaired H_2_S generation or consumption. Overall, spatial coordination of sulfide metabolism across compartments is essential for plant resilience and integrates redox signaling and autophagy to mitigate environmental stress. These findings suggest an approach to develop crops with improved stress tolerance by regulating the balance of gasotransmitter within specific organelles.

## 5 Environmental implication

Cadmium contamination in agricultural soils threatens global food security by impairing plant physiology, reducing crop yield and quality, and posing serious health risks through food-chain accumulation. Strengthening the protective mechanisms of crops is essential for mitigating cadmium stress. Improving tolerance, such as by modulating H_2_S signaling, can sustain productivity in contaminated soils and reduce cadmium levels in edible tissues, safeguarding human health. The findings from this research are crucial for developing biotechnological strategies that increase plant defense responses to environmental stress. This knowledge will guide future applications aimed at improving crop resilience, supporting the economy, and ensuring long-term food security.

## CRediT authorship contribution statement

**Reyes Carrillo:** Data curation, Formal analysis, Investigation, Methodology, Visualization. **Inmaculada Moreno:** Formal analysis, Investigation, Methodology. **Angeles Aroca:** Conceptualization, Data curation, Formal analysis, Funding acquisition, Resources, Validation, Writing – original draft, Writing – review & editing. **Cecilia Gotor:** Conceptualization, Data curation, Formal analysis, Funding acquisition, Project administration, Resources, Validation, Writing – original draft, Writing – review & editing.

## Declaration of competing interest

The authors declare that they have no known competing financial interests or personal relationships that could have appeared to influence the work reported in this paper.

## Data Availability

Data will be made available on request.
