# Supplementary material for: Organelle-specific hydrogen sulfide metabolism governs redox homeostasis to regulate plant autophagy and cadmium stress resilience
Source: Redox Biol. 2026 Apr 17;93:104177. doi: 10.1016/j.redox.2026.104177 (PMC13122702; doi:10.1016/j.redox.2026.104177)
Supplement: Multimedia component 1 [file mmc1.pptx]

## Slide 1
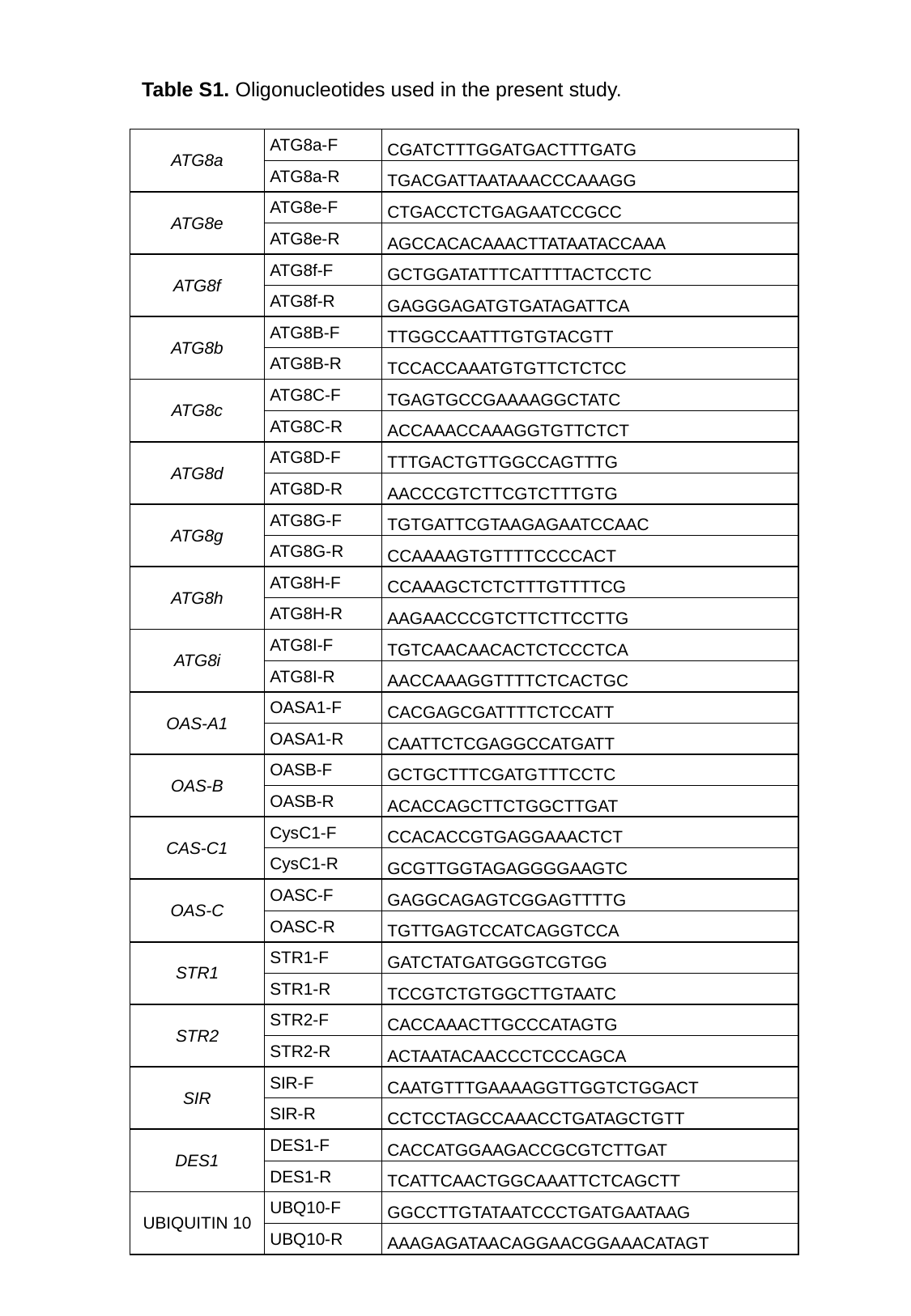

Table S1. Oligonucleotides used in the present study.
| ATG8a | ATG8a-F | CGATCTTTGGATGACTTTGATG |
| --- | --- | --- |
| | ATG8a-R | TGACGATTAATAAACCCAAAGG |
| ATG8e | ATG8e-F | CTGACCTCTGAGAATCCGCC |
| | ATG8e-R | AGCCACACAAACTTATAATACCAAA |
| ATG8f | ATG8f-F | GCTGGATATTTCATTTTACTCCTC |
| | ATG8f-R | GAGGGAGATGTGATAGATTCA |
| ATG8b | ATG8B-F | TTGGCCAATTTGTGTACGTT |
| | ATG8B-R | TCCACCAAATGTGTTCTCTCC |
| ATG8c | ATG8C-F | TGAGTGCCGAAAAGGCTATC |
| | ATG8C-R | ACCAAACCAAAGGTGTTCTCT |
| ATG8d | ATG8D-F | TTTGACTGTTGGCCAGTTTG |
| | ATG8D-R | AACCCGTCTTCGTCTTTGTG |
| ATG8g | ATG8G-F | TGTGATTCGTAAGAGAATCCAAC |
| | ATG8G-R | CCAAAAGTGTTTTCCCCACT |
| ATG8h | ATG8H-F | CCAAAGCTCTCTTTGTTTTCG |
| | ATG8H-R | AAGAACCCGTCTTCTTCCTTG |
| ATG8i | ATG8I-F | TGTCAACAACACTCTCCCTCA |
| | ATG8I-R | AACCAAAGGTTTTCTCACTGC |
| OAS-A1 | OASA1-F | CACGAGCGATTTTCTCCATT |
| | OASA1-R | CAATTCTCGAGGCCATGATT |
| OAS-B | OASB-F | GCTGCTTTCGATGTTTCCTC |
| | OASB-R | ACACCAGCTTCTGGCTTGAT |
| CAS-C1 | CysC1-F | CCACACCGTGAGGAAACTCT |
| | CysC1-R | GCGTTGGTAGAGGGGAAGTC |
| OAS-C | OASC-F | GAGGCAGAGTCGGAGTTTTG |
| | OASC-R | TGTTGAGTCCATCAGGTCCA |
| STR1 | STR1-F | GATCTATGATGGGTCGTGG |
| | STR1-R | TCCGTCTGTGGCTTGTAATC |
| STR2 | STR2-F | CACCAAACTTGCCCATAGTG |
| | STR2-R | ACTAATACAACCCTCCCAGCA |
| SIR | SIR-F | CAATGTTTGAAAAGGTTGGTCTGGACT |
| | SIR-R | CCTCCTAGCCAAACCTGATAGCTGTT |
| DES1 | DES1-F | CACCATGGAAGACCGCGTCTTGAT |
| | DES1-R | TCATTCAACTGGCAAATTCTCAGCTT |
| UBIQUITIN 10 | UBQ10-F | GGCCTTGTATAATCCCTGATGAATAAG |
| | UBQ10-R | AAAGAGATAACAGGAACGGAAACATAGT |
