# Supplementary material for: Organelle-specific hydrogen sulfide metabolism governs redox homeostasis to regulate plant autophagy and cadmium stress resilience
Source: Redox Biol. 2026 Apr 17;93:104177. doi: 10.1016/j.redox.2026.104177 (PMC13122702; doi:10.1016/j.redox.2026.104177)
Supplement: Multimedia component 2 [file mmc2.pptx]

## Slide 1
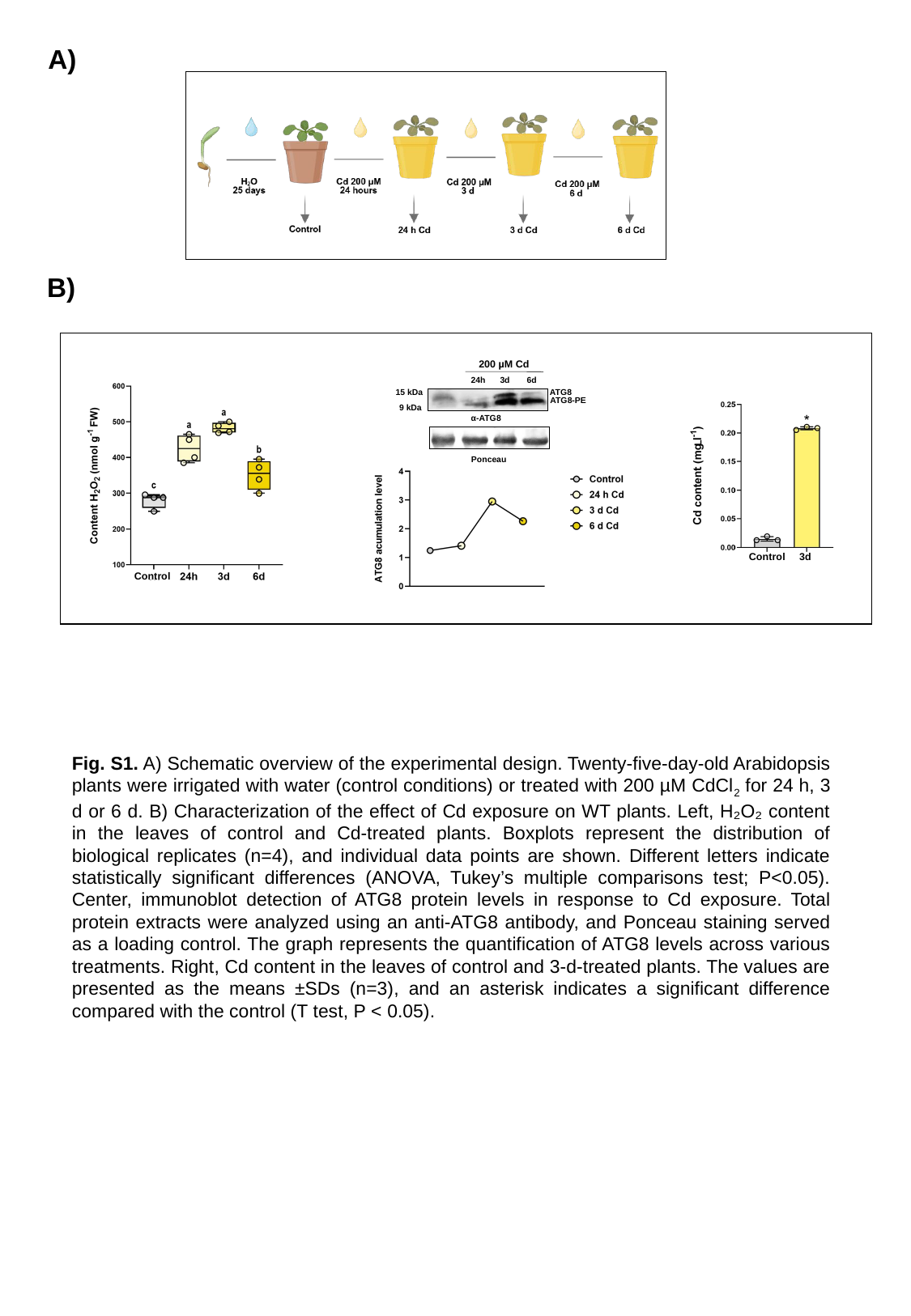

A)
B)
200 µM Cd
24h 3d 6d
ATG8
15 kDa
ATG8-PE
9 kDa
α-ATG8
Ponceau
Control
Control 3d
L
Fig. S1. A) Schematic overview of the experimental design. Twenty-five-day-old Arabidopsis plants were irrigated with water (control conditions) or treated with 200 µM CdCl2 for 24 h, 3 d or 6 d. B) Characterization of the effect of Cd exposure on WT plants. Left, H₂O₂ content in the leaves of control and Cd-treated plants. Boxplots represent the distribution of biological replicates (n=4), and individual data points are shown. Different letters indicate statistically significant differences (ANOVA, Tukey’s multiple comparisons test; P<0.05). Center, immunoblot detection of ATG8 protein levels in response to Cd exposure. Total protein extracts were analyzed using an anti-ATG8 antibody, and Ponceau staining served as a loading control. The graph represents the quantification of ATG8 levels across various treatments. Right, Cd content in the leaves of control and 3-d-treated plants. The values are presented as the means ±SDs (n=3), and an asterisk indicates a significant difference compared with the control (T test, P < 0.05).
