# Supplementary material for: Organelle-specific hydrogen sulfide metabolism governs redox homeostasis to regulate plant autophagy and cadmium stress resilience
Source: Redox Biol. 2026 Apr 17;93:104177. doi: 10.1016/j.redox.2026.104177 (PMC13122702; doi:10.1016/j.redox.2026.104177)
Supplement: Multimedia component 3 [file mmc3.pptx]

## Slide 1
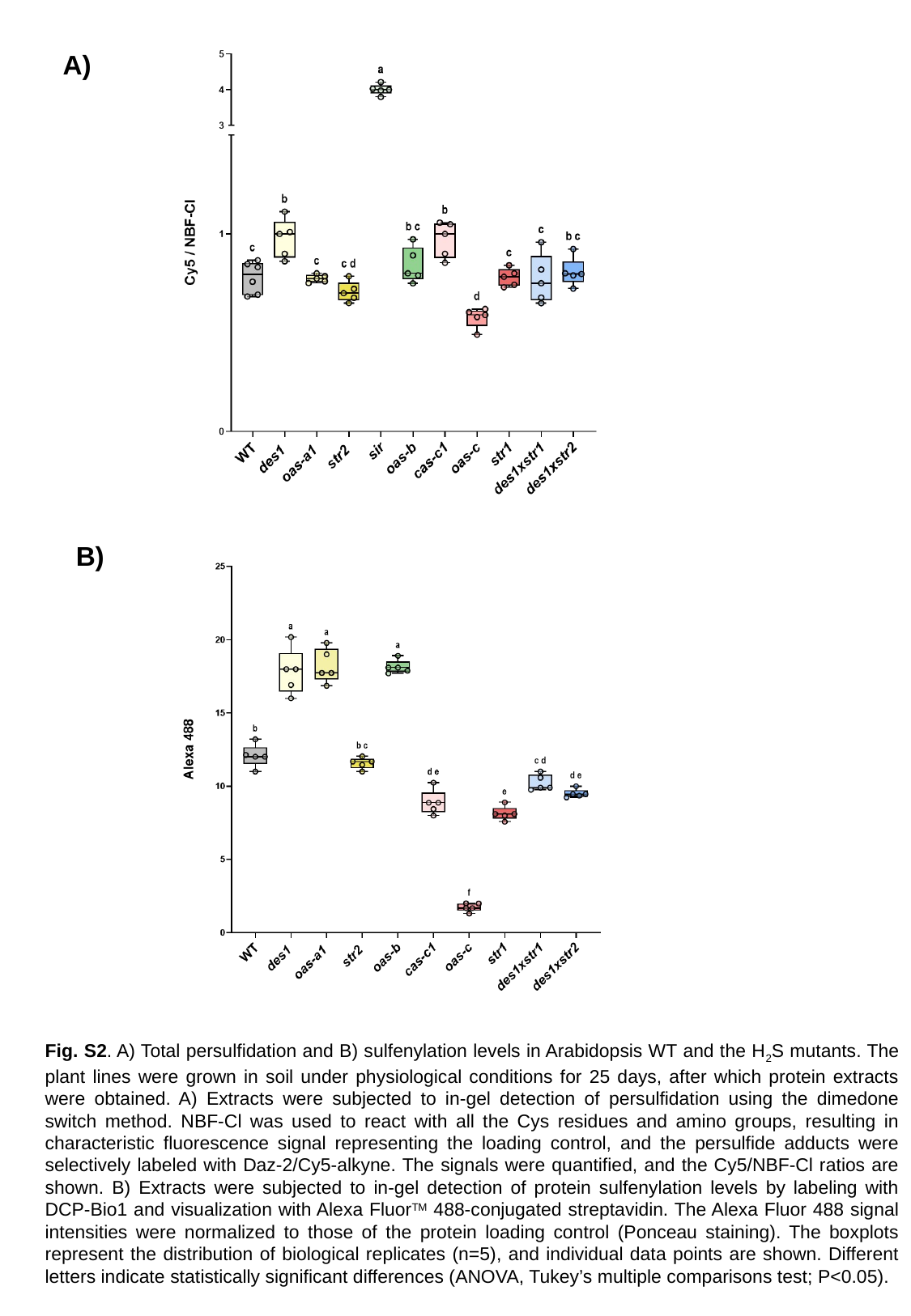

A)
B)
Fig. S2. A) Total persulfidation and B) sulfenylation levels in Arabidopsis WT and the H2S mutants. The plant lines were grown in soil under physiological conditions for 25 days, after which protein extracts were obtained. A) Extracts were subjected to in-gel detection of persulfidation using the dimedone switch method. NBF-Cl was used to react with all the Cys residues and amino groups, resulting in characteristic fluorescence signal representing the loading control, and the persulfide adducts were selectively labeled with Daz-2/Cy5-alkyne. The signals were quantified, and the Cy5/NBF-Cl ratios are shown. B) Extracts were subjected to in-gel detection of protein sulfenylation levels by labeling with DCP-Bio1 and visualization with Alexa FluorTM 488-conjugated streptavidin. The Alexa Fluor 488 signal intensities were normalized to those of the protein loading control (Ponceau staining). The boxplots represent the distribution of biological replicates (n=5), and individual data points are shown. Different letters indicate statistically significant differences (ANOVA, Tukey’s multiple comparisons test; P<0.05).
